# A rare case of pituicytoma-related hyperprolactinemia due to mass effect on infundibular stalk—Case report

**DOI:** 10.1016/j.ijscr.2023.108348

**Published:** 2023-05-24

**Authors:** Mohammed Maan Al-Salihi, Alaaeldin Ahmed, Maryam Sabah Al-Jebur, Yezan Al-Salihi, Md Moshiur Rahman, Ali Ayyad

**Affiliations:** a Department of Neurosurgery, Hamad General Hospital, Doha, Qatar; b Department of Neurological Surgery, School of Medicine and Public Health, University of Wisconsin, Madison, WI, USA; c College of Medicine, University of Baghdad, Baghdad, Iraq; d Neurosurgery Department, Holy Family Red Crescent Medical College, Dhaka, Bangladesh; e Department of Neurosurgery, Saarland University Hospital, Homburg, Germany

**Keywords:** Pituicytoma, Pituitary tumors, Low-grade glial tumors, Immunohistochemical studies, Craniotomy

## Abstract

**Introduction and importance:**

Pituicytomas are extremely rare cancers of the sellar and suprasellar region that appear from the infundibulum or posterior pituitary. World Health Organization in 2007, described pituicytoma as a low-grade tumour (Grade I) in the taxonomy of CNS cancers. The tumour can frequently simulate a pituitary adenoma and is also linked with hormonal disorders. Distinguishing a pituitary adenoma from a pituicytoma can be challenging. We present a rare case report where an elderly female showed high levels of prolactin mainly due to mass effects along with diagnostic, imaging, and immunohistochemical characteristics of pituicytoma.

**Case presentation:**

A 50-year-old female known case of hypothyroidism, complained of headache associated with dizziness and blurry vision. Her prolactin levels were high which led to the suspicion of pituitary involvement and underwent MRI. The imaging study revealed a well-defined, completely suprasellar, homogenously enhancing mass lesion arising from the left lateral aspect of the pituitary infundibulum. The initial differential diagnosis from the imaging included an ectopic pituitary gland, adenoma, pituicytoma, or hypothalamic glioma. She underwent a right supra-orbital craniotomy for debulking of the pituitary stalk lesion. The histopathological diagnosis was pituicytoma, WHO grade I.

**Clinical discussion:**

The clinical manifestations are mostly depended upon the tumour mass and position. They typically present due to mass effects leading to hormonal disorders. The imaging studies are the backbone of the clinical diagnosis along with the histopathological findings. Surgical resection is the preferred treatment for pituicytoma, with an exceptionally low recurrence rate (4.3 %) following complete removal.

**Conclusion:**

Pituicytomas are slow-growing, benign glial growths. It is challenging to diagnose before surgery as its clinical manifestations and imaging findings look like those of non-functional pituitary adenomas. The effective treatment for pituicytoma is gross total resection by the endoscopic method or transcranial technique.

## Highlights

• Pituicytomas are slow-growing, benign glial growths.
• The clinical manifestations are mostly depended upon the tumor mass and position.
• The typical presentation of pituicytomas is progressive mass effect symptoms.
• Postoperative complications are mostly due to iatrogenic injury to connecting structures.

## 1 Introduction

Pituicytomas are extremely rare cancers of the sellar and suprasellar region that appear from the infundibulum or posterior pituitary. In 1955, this tumour was first recognized in the literature, and a total of 142 cases of pituicytoma have been stated to date [1,2]. World Health Organization in 2007, described pituicytoma as a low-grade tumour (Grade I) in the taxonomy of CNS cancers [3]. The tumour can often simulate a pituitary adenoma and is also linked with hormonal disorders [4,5]. Other clinical manifestations involve compression of the optic chiasm, infundibulum or posterior pituitary gland causing blurring of vision; headache; and characteristics of hypopituitarism such as fatigue, mildly elevated serum prolactin, menstrual irregularities and decreased libido [6]. The tumour is typically a dense and defined mass lesion of the sellar and suprasellar spaces on magnetic resonance imaging (MRI). Frequently, it is isointense to grey matter on T1-weighted images, hyperintense on T2-weighted images and homogeneously contrast enhancing. Typically, pituicytoma was connected with hyperactivity of the adjacent pituitary glandular tissue, which resulted in clinically irregular hormone secretion. The diagnosis of pituicytoma is challenging when the patients present with hormone dysfunction [7]. Though, diagnosis is classically made based on histopathological findings and immunohistochemical (IHC) studies. These tumors are managed by surgical resection with a good prognosis. Here, we present a case of pituicytoma with hyperprolactinemia and headache reported rarely in the existing clinical literature.

## 2 Presentation of case

We present the case of a 50-year-old lady with a known history of hypothyroidism who presented to the Emergency Department with a three-month history of headaches associated with dizziness and blurry vision. The headache was mainly involving the entire head, without any diurnal variation, and was not associated with nausea, vomiting, photophobia, neck pain or stiffness, or any new behavioral changes. Upon further inquiry, the patient reported menstrual irregularities, which led to medical attention. On examination, the patient had a Glasgow Coma Scale (GCS) score of 15 with no other neurological deficits.

**Fig. 1:**
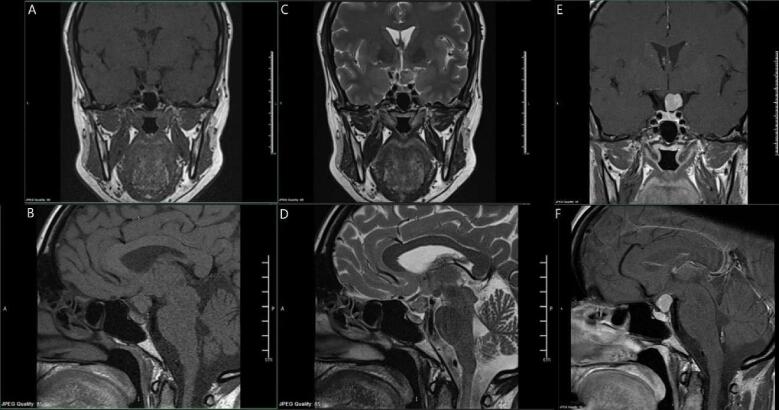
Preoperative MRI, A & B are T1 MRI, C&D are T2 MRI, E & F are T1 MRI with contrast, showing A well-defined completely suprasellar homogenously enhancing mass lesion is seen arising from the left lateral aspect of the pituitary infundibulum abutting the left optic tract and tuber cinereum measuring approximately 1.1 × 1.2 cm. Pituitary gland is seen separately. The lesion shows intermediate signal intensity on T1 and T2-weighted images.

Laboratory investigations revealed high prolactin levels (1346 mIU/L), prompting an MRI pituitary scan with a suspicion of a pituitary adenoma. The imaging showed a well-defined, completely suprasellar, homogenously enhancing mass lesion arising from the left lateral aspect of the pituitary infundibulum, abutting the left optic tract and tuber cinereum, measuring approximately 1.1 × 1.2 cm. The pituitary gland was seen separately. The lesion showed intermediate signal intensity on T1 and T2-weighted images (Fig. 1). The initial differential diagnosis from the imaging included an ectopic pituitary gland with either hyperplasia of TSH-secreting cells secondary to hypothyroidism (with associated hyperprolactinemia due to mass effect on a stalk), adenoma, pituicytoma, or hypothalamic glioma.

**Fig. 2:**
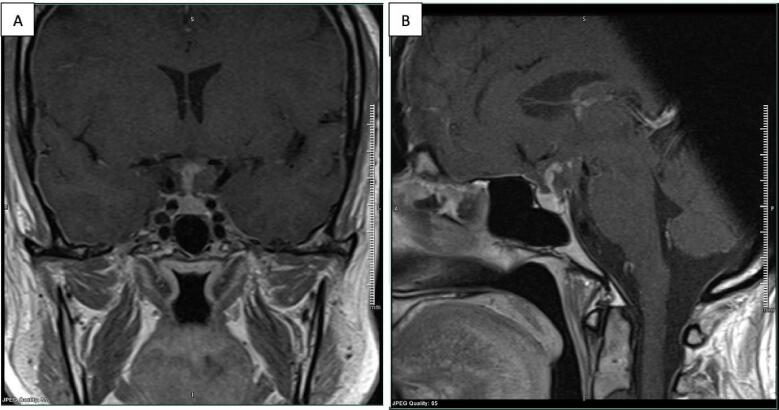
Postoperative MRI findings. Adjacent expected post-operative changes are seen. A small, L-shaped residual enhanced part of the mass is still seen. B It represents the superior and right anterolateral aspect of the mass.

**Fig. 3:**
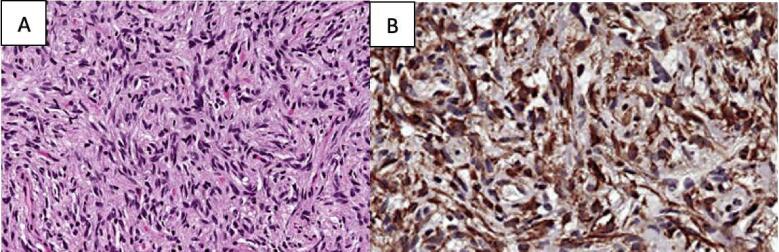
Pituicytoma. A The histology varies from streaming bundles of cells to a somewhat storiform pattern. B Strong staining for glial fibrillary acidic protein is seen.

The patient underwent a right supra-orbital craniotomy for debulking of the pituitary stalk lesion (Fig. 2). Final histopathology showed marginal pleomorphism with a tumour containing spindle-shaped cells, which is separated by a storiform structure (Fig. 3). Mitotic activity was not significant. The tumour was immunoreactive for S-100 protein, glial fibrillary acidic protein (GFAP), vimentin and epithelial membrane antigen (EMA) on immunohistochemical studies.

Postoperatively, the patient developed third nerve palsy, which improved daily until the discharge date. Two weeks after surgery, the palsy had completely resolved.

**Fig. 4:**
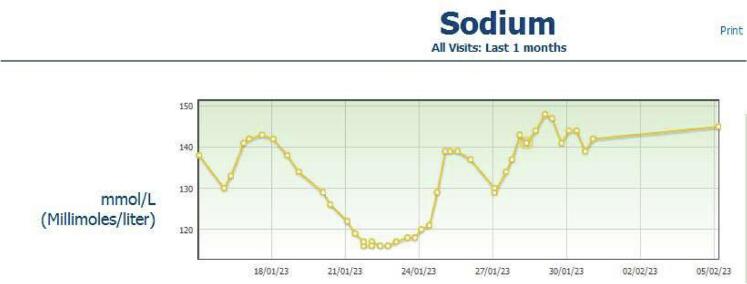
SIADH. Laboratory results of sodium levels in mmol/L during one month.

Additionally, the patient developed signs of the syndrome of inappropriate antidiuretic hormone secretion (SIADH), with low sodium levels (129 mmol/L), during her hospital course (Fig. 4), which extended for almost two weeks post-operatively. However, with fluid restrictions and sodium replacement therapy, the patient's sodium levels eventually returned to normal and the symptoms resolved. This case report followed the SCARE guidelines for its realization [8].

## 3 Discussion

The term pituicytoma was first coined in 1958 [9]. It is also known as pituicytoma, choristoma, granular cell tumour, infundibuloma, myoblastoma, pilocytic astrocytoma and posterior pituitary astrocytoma in clinical databases. MRI features were given by Hurley in 1994 which explained them as nonpilocytic cancer [10]. In 2000, Brat et al. proposed the pathological criteria to diagnose pituicytoma [11]. World Health Organization (WHO) 2007 classification included it as a sellar astrocytic neoplasm which is a benign grade I tumour. It was independently categorized as a grade I tumour among cancers of the sellar region in the 2016 and 2017 WHO classifications of cancers of CNS [12,13]. Regarding the epidemiology of pituicytomas, numerous case reports assessed the clinical literature and discovered that gender predilection was not seen in these tumors. Total of 142 cases reported to date in the literature. These cases were equally distributed (ratio 1:1) among males and females. The mean age of these cases was 47.2 years, ranging between 10 and 85 years. Imaging findings were accessible in 120 cases only. The mean size of the tumour was 25 mm (ranging from 5 to 74 mm). These tumors were mostly identified extending in a suprasellar cistern (35.4 %) or pure sellar region (22.3 %) and/or in the cavernous sinuses (8.2 %) [2].

The clinical manifestations are usually due to the local effects of growth, and consequently, depending on the growing mass and its position. For example, hypopituitarism and headache are caused by hypophysis compression; blurring of vision and visual disturbances are caused by optic chiasm compression and hypothalamic dopamine hormonal disturbances are due to infundibular compression which can lead to hyperprolactinemia, hyposexuality, sexual disorders and amenorrhea [1]. Chu et al. reported a 45 yrs. woman with hyperprolactinemia and thickening of the pituitary stalk similar to the case in our study. Pituicytoma presented as hyperprolactinemia is rarely reported [14]. In our case in this study, presented with combinations of blurring of vision, headache, dizziness, and menstrual irregularities which is consistent with the earlier reported cases. Few studies have reported other hormonal disturbances in patients diagnosed with pituicytoma such as Cushing syndrome as found through a detailed PubMed literature search. These hormonal defects are mainly due to a mass effect [4,5,[15], [16], [17], [18], [19]].

On MRI imaging, after administration of gadolinium, pituicytomas are identified as isointense on T1 weighted images and T2 weighted images are hyperintense, with noticeable homogeneous development [20]. Gibbs et al. [21] showed early and late staining patterns of the tumour throughout the venous phase within the selected internal carotid artery visible on angiograms. The late venous phase clearly showed the outline of the tumour stain so, we can conclude that pituicytoma had a vaguely late enhancement on dynamic contrast studies on contrary, pituitary adenoma was seen during the earlier arterial phase. The tumour growth showed blood vessel networks on histology assessment with heterogeneous development, cystic change, and calcification were hardly visible [22].

Surgical resection is the preferred treatment for pituicytoma, with an exceptionally low recurrence rate (4.3 %) following complete removal. A single case showed tumour recurrence among all the described cases in literature treated with gross total resection (GTR) [23]. Recent conventional surgical methodologies include the aforesaid transsphenoidal and frontotemporal craniotomy. Complete resection of recurring pituicytomas was suggested by Feng et al. [24] when the surgical resection was done through various techniques such as endonasal, transsphenoidal, expanded endoscopic and transplanum. But, the safety and efficiency of the expanded transsphenoidal technique are still not completely recognized as there is presently inadequate clinical data. Postoperatively, Diabetes insipidus [25,26], visual impairment, hypopituitarism, and hypothyroidism are considered to be the most common complications [27]. These complications are mostly due to the iatrogenic injury to the connecting structures. Our patient had developed SIADH after the surgical resection and debulking. She developed hyponatremia which was managed accordingly and was discharged after two weeks leading to prolong hospital stay. Hussein et al. reported that Hyponatremia is a fairly common event following the transsphenoidal operation, and is linked with prolonged hospital stay and the possibility of readmission. The usefulness of fluid restriction is limited in these cases [26,28].

## 4 Conclusion

Pituicytoma arises from the sellar and suprasellar regions and is considered a rare WHO grade I cancer, which can manifest with diverse clinical, imaging, and hormonal characteristics, similar to pituitary adenomas. Histopathological examination and Immunohistochemical studies form the mainstay in diagnosis. Surgical removals extending from biopsy to gross total resection (GTR) have been explained, with the transsphenoidal approach being widely performed. Postoperative complications included mainly diabetes insipidus, visual disturbances, hypothyroidism and hypopituitarism. These complications are mostly due to the iatrogenic injury to the connecting structures. The prognosis from the available literature seems to be good in the majority of the cases after surgery alone or in combination with radiotherapy.

## Patient consent

Written informed consent was obtained from the patient for the publication of this case report and accompanying images. A copy of the written consent is available for review by the Editor-in-Chief of this journal on request.

## Ethical approval

Baghdad hospital exempts ethical approval and supply the informed written consent for this case report.

## Funding

None.

## Author contribution

All authors equally contributed to the analysis and writing of the manuscript.

## Guarantor

Md Moshiur Rahman

## Research registration number

N/a.

## Conflict of interest statement

None.
